# *MTHFR* c.677C>T Inhibits Cell Proliferation and Decreases Prostate Cancer Susceptibility in the Han Chinese Population in Shanghai

**DOI:** 10.1038/srep36290

**Published:** 2016-11-07

**Authors:** Jun-Long Wu, Shu-Xian Zhou, Rui Zhao, Xuan Zhang, Kun Chang, Cheng-Yuan Gu, Hua-Lei Gan, Bo Dai, Yao Zhu, Hai-Liang Zhang, Guo-Hai Shi, Yuan-Yuan Qu, Jian-Yuan Zhao, Ding-Wei Ye

**Affiliations:** 1 Department of Urology, Fudan University Shanghai Cancer Center, Shanghai 200032, China; 2 Department of Oncology, Shanghai Medical College, Fudan University, Shanghai 200032, China; 3 The State Key Laboratory of Genetic Engineering and Collaborative Innovation Center of Genetics & Development, School of Life Sciences, Fudan University, Shanghai 200433, China; 4 Institutes of Biomedical Sciences, Fudan University, Shanghai 200032, China; 5 Department of Pathology, Fudan University Shanghai Cancer Center, Shanghai 200032, China

## Abstract

Methylenetetrahydrofolate reductase (*MTHFR*) c.677C>T and c.1298A>C variants were known to be associated with prostate cancer (PCa) risk with conflicting results, because of *MTHFR* and nutrient status interaction in the prostate development. In this large-scale, hospital-based, case-control study of 1817 PCa cases and 2026 cancer-free controls, we aimed to clarify the association between these two *MTHFR* variants and PCa risk in Shanghai and to explore the underlying molecular mechanisms. We found that both the heterozygous CT (adjusted OR = 0.78, 95% CI: 0.67–0.92) and the homozygous TT genotypes (adjusted OR = 0.68, 95% CI: 0.55–0.83) of c.677C>T were associated with a significantly decreased risk of PCa compared with homozygous wild-type CC genotype, respectively, using multivariate logistic regression. Furthermore, we confirmed that *MTHFR* c.677T allele was related to an increased serum homocysteine level in the Han Chinese population in Shanghai. In the cultured PCa cell lines, we observed that *MTHFR* c.677T could elevate the cellular homocysteine level and cause DNA damage, thus increasing cell apoptosis and finally inhibiting cell proliferation. In conclusion, *MTHFR* c.677T was a protective factor of PCa risk in ethnic Han Chinese males by inducing DNA damage and cell apoptosis.

Prostate cancer (PCa) is the second most frequently diagnosed cancer and the fifth leading cause of cancer death worldwide among men, and remains the first frequently diagnosed cancer in more developed countries^1^. Although PCa incidence is much lower than in Western countries, it is still in the top 10 most common cancers in Chinese males^2^. The increased incidence rate of PCa in China was due to prolonged life expectancy, dietary changes, more PSA testing and Westernized lifestyle^3^. The PCa age-standardized incidence (12.96 per 100,000) and age-standardized mortality (4.97 per 100,000) in Shanghai were the highest among all provinces of China^4^. This phenomenon aroused many researchers’ interest. Higher economic development level, better medical level and being more receptive to western culture, lifestyle and diet may partly explain the difference^5^, but specific connections remained unknown.

PCa is a complex disease; and many etiological factors, including genetic profile, nutrition, environmental exposures, etc., are thought to play important roles in cancer development^6^. It is widely acknowledged that genetic polymorphisms may act as a predictors of some characteristics of PCa, including incidence rate^7^,^8^,^9^, pathology^10^, progression^11^, etc.

Folates are cofactors and cosubstrates for biological methylation and nucleic acid synthesis and also act as regulatory molecules. The folate-mediated, one-carbon metabolic pathway is fundamental to DNA synthesis, repair and methylation^12^; and plays an important role in many types of diseases, such as cancers, birth defects and cardiovascular diseases^13^. Over the past decades, a series of clinical studies showed that the concentration of homocysteine, folate and vitamin B12—the key metabolites in the folate pathway—were related to the occurrence of multiple types of cancers, including PCa^14^, breast cancer^15^, and gastric cancer^16^. Numerous studies reported that increasing plasma homocysteine level was associated with a reduced PCa risk^17^,^18^. Thus, extensive analyses of genes involved in the folate metabolism pathway have been conducted. The methylenetetrahydrofolate reductase (*MTHFR*) gene encodes an enzyme that catalyzes the irreversible conversion of 5′,10′-methylenetetrahydrofolate to 5-methyltetrahydro folate, which serves as a methyl donor in the remethylation of homocysteine to methionine. Until now, the *MTHFR* gene has been identified to possess 98 different variants with benign, pathogenic or unknown significance which are listed on ClinVar database (http://www.ncbi.nlm.nih.gov/clinvar/). Among these variants, rs1801133 (c. 677C>T) and rs1801131 (c.1298A>C) are two most extensively reported polymorphisms with a global minor allele frequency (GMAP) 0.245 and 0.249, respectively^19^,^20^. These two variants have shown to be associated with the risk of PCa, although somewhat conflicting results have been reported^21^,^22^,^23^,^24^.

Wu *et al*. reported a significant reduction of PCa risk in yellow people who carried c.677T variant in Taiwan^25^, and many groups achieved similar results in other ethnic regions^26^,^27^. However, a recent meta-analysis found no significant association between the those variants and PCa risk^21^, and subgroup analysis indicated that the association between variants and PCa risk may vary among ethnic groups. In this meta-analysis, only two studies enrolled focused on the ethnic Han Chinese population. Wu *et al*. performed study in Taiwan while Cai *et al*. performed study in the Liaoning Province, both far different regions from Shanghai. There was a limited sample size in the previous study regarding *MTFHR* variants in Han Chinese PCa susceptibility; and more importantly, this association has not been surveyed in Shanghai city, which had the highest PCa incidence in China. The current study aimed to investigate the association between these two common variants of *MTHFR* and PCa risk in a large-scale, hospital-based, case-control cohort in Shanghai city and to explore the underlying molecular mechanisms.

## Results

### Characteristics of the study population

The demographic characteristics of the 1817 PCa patients and 2026 normal controls were presented in Table 1. Since we performed a frequency match between PCa patients and cancer-null controls, the age of diagnosis between the two groups were well matched with a mean age of 66.7 in the PCa patients group and a mean age of 66.9 in the control group. Besides, there were no significant differences in the distribution between the two groups in the following indexes: BMI, hypertension, diabetes mellitus, and cardiovascular diseases. Apparently, serum PSA level was significantly higher in the PCa patients group (28.5 ng/ml) due to the nature of PCa and the filtration we performed before enrollment. Among all PCa patients, 605 patients (33.3%) had a Gleason score ≥ 8, 526 patients (32.2%) had extracapsular extension, 154 patients (8.5%) had lymph node involvement and 352 patients (19.4%) had positive surgical margins.

### Association between two *MTHFR* gene single nucleotide polymorphisms (SNPs) and PCa risk

The genotype frequencies and their associations with the risk of PCa were summarized in Table 2. All the genotype frequencies of the two selected *MTHFR* gene SNPs among controls met the criteria of Hardy-Weinberg Equilibrium (all *P* > 0.05). We found a significantly different genotype frequency distribution of rs1801133 (C677T) between the PCa group and the cancer-free group (*P* < 0.001), while no different genotype frequency distribution of rs1801131 (A1298C) was observed between cases and controls (*P* = 0.690). Both the heterozygous CT genotype (crude OR = 0.79, 95% CI: 0.68–0.91) and the homozygous TT genotype (crude OR = 0.65, 95% CI: 0.54–0.78) of rs1801133 were associated with a significantly decreased risk of PCa, compared with homozygous wild-type CC genotype, respectively. In addition, the relationship between the two *MTHFR* genotypes and the risk of PCa was evaluated using dominant models, recessive models and additive models, respectively, with all three models achieving statistical significance; which indicated that people who carried T allele of rs1801133 had a lower risk of developing PCa. Besides, no clear association between genotype frequency of rs1801131 and PCa risk was observed. Furthermore, we used multivariate logistic regression analyses to reanalyze the above-mentioned genotypes after adjusting for age at diagnosis, BMI, hypertension, diabetes mellitus, and cardiovascular disease. We found that all of the genotypes maintained the same relationship with PCa risk as declared above in a univariate logistic regression model. Finally, we found a 32% decreased risk of PCa for people who carried homozygous TT genotype of rs1801133 (adjusted OR = 0.68, *P* < 0.001) compared with those who carried homozygous wild-type CC genotype.

### Stratification analysis

We performed a stratified analysis to evaluate the association between SNPs mentioned above and PCa risk in different subgroups using a recessive model. Analytic results were presented in Table 3. In multivariate logistic regression analyses, we found that the homozygous TT genotype of rs1801133 was significantly associated with a decreased risk of PCa risk in subgroups of patients with a Gleason score ≥8, patients with extracapsular extension, patients with seminal vesicle invasion, and patients with or without lymph node involvement. The results were all further supported by a homogeneity test using the χ^2^-based Q test (all *P* < 0.05). Besides, a decreased risk of PCa was observed among subgroups age >68 years, a BMI of <25 kg/m^2^, with hypertension, without diabetes mellitus and without cardiovascular disease for patients carrying *MTHFR* rs1801133 TT genotype; and an increased risk of PCa was observed among subgroups with cardiovascular disease with lymph node involvement and those carrying the *MTHFR* rs1801131 CC genotype, further homogeneity tests didn’t support the differences in PCa risk found between these strata.

### Variants of *MTHFR* alter plasma and cellular homocysteine level

**Figure 1:**
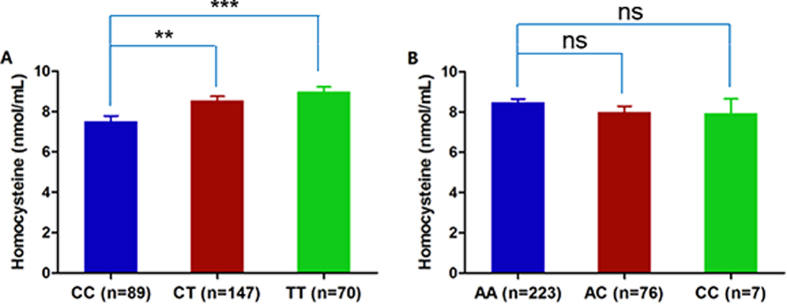
*MTHFR* c.677T was related to homocysteine level in the Han Chinese population. (**A**) Both heterozygous CT genotype and homozygous TT genotype of rs1801133 are significantly related with a higher serum homocysteine level. (**B**) Neither heterozygous AC genotype nor homozygous CC genotype of rs1801131 is related with a serum homocysteine level. **Indicates *P* < 0.01, ***indicates *P* < 0.001.

**Figure 2:**
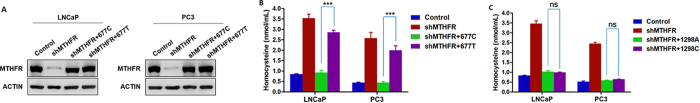
*MTHFR* c.677T variant leads to a notably increased cellular homocysteine level in PCa cell lines. (**A**) Knockdown efficiency of *shMTHFR* and protein expression of MTHFR after restoration of wild-type or mutant *MTHFR* gene was measured by western blot. The full-length blots were displayed in Supplementary Figure 1. (**B**) c.677T variant of *MTHFR* gene leads to an increased cellular homocysteine level in both LNCaP and PC3 cell lines. (**C**) c.1298C variant of *MTHFR* gene has no effect on the cellular homocysteine level in LNCaP and PC3 cell lines. ***Indicates *P* < 0.001, ns indicates no significance.

MTHFR is an essential enzyme in one-carbon metabolism, producing methyl donor for the conversion of homocysteine to methionine. Thus, variants of *MTHFR* may alter enzymatic function and change the serum homocysteine level. To test this hypothesis, we randomly selected 306 participants from a control group and detected the serum homocysteine levels. Among the 306 participants, 89 (29.1%) carried the wild-type CC genotype of rs1801133, 147 (48.0%) carried the heterozygous CT genotype and 70 (22.9%) carried the homozygous TT genotype. We found that people with the heterozygous CT genotype or homozygous TT genotype had a significantly higher serum homocysteine level compared with wild-type CC carriers (Fig. 1A). However, both heterozygous AC genotype and homozygous CC genotype of rs1801131 had no significant effects on plasma homocysteine concentration (Fig. 1B). Furthermore, we set up *MTHFR*-knockdown LNCaP and PC3 cell lines which were validated by western blotting (Fig. 2A) and tested cellular homocysteine level. It showed that both in LNCaP and PC3 cell lines, homocysteine level in *MTHFR*-knockdown cells was significantly higher than in control cells (Fig. 2B,C). Then, we overexpressed c.677C *MTHFR*, c.677T *MTHFR*, c.1298A *MTHFR* and c.1298C *MTHFR*, respectively, in *MTHFR*-knockdown LNCaP and PC3 cell lines. We found that that the cellular homocysteine level of c.677T *MTHFR*-overexpressed cells was significantly higher than that of wild-type *MTHFR*-overexpressed cells (Fig. 2B). However, we did not observe a significant difference in cellular homocysteine levels between c.1298C *MTHFR*-overexpressed cells and wild-type *MTHFR*-overexpressed cells (Fig. 2C).

### *MTHFR* c.677T causes DNA damage

**Figure 3:**
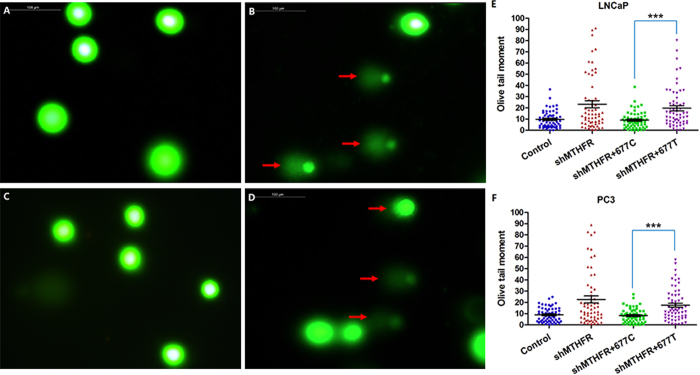
*MTHFR* c.677T variant causes DNA damage. (**A**) Comet Assay results in control cells. (**B**) Comet Assay results in *MTHFR*-knockdown cells, indicating that down-regulation of *MTHFR* leads to DNA damage. (**C**) Comet Assay results in *MTHFR*-knockdown + wild-type overexpressed cells, indicating that overexpression of 677C-*MTHFR* can reduce DNA damage. (**D**) Comet Assay results in *MTHFR*-knockdown + 677T-*MTHFR* overexpressed cells, indicating that overexpression of 677T-*MTHFR* cannot reduce DNA damage. (**E**) Quantitative Comet Assay results in LNCaP cells, indicating that c.677T variant might cause DNA damage compared with wild-type *MTHFR*. (**F**) Same results are achieved in PC3 cell lines. Red arrow points out the tails, which indicate DNA damage.

We performed Comet Assay to assess DNA damage. In normal cells, the fluorescence is confined mostly to the nucleus because undamaged DNA cannot migrate. In cells with DNA damage, DNA is denatured by the alkali solution used for single-strand break detection or the neutral solution used for double-strand break detection. The negatively charged DNA fragments are then released from the nucleus and migrate toward the anode. The results of control cells and *MTHFR*-knockdown cells were presented in Fig. 3A,B. The results of wild-type *MTHFR*-overexpression and c.677T *MTHFR*-overexpression in *MTHFR*-knockdown cell lines were presented in Fig. 3C,D. It showed that *MTHFR* knockdown could lead to DNA damage. Overexpression of wild-type *MTHFR* in these cells could repair DNA damage, but overexpression of c.677T *MTHFR* could not. Quantitative Comet Assay results were presented in Fig. 3E,F, which showed that c.677T variant of *MTHFR* could lead to DNA damage (*P* < 0.001). Overexpression of c.1298A and c.1298C *MTHFR* in *MTHFR*-knockdown cell lines both could repair DNA damage (data not shown).

### *MTHFR* c.677T variant increases cell apoptosis

**Figure 4:**
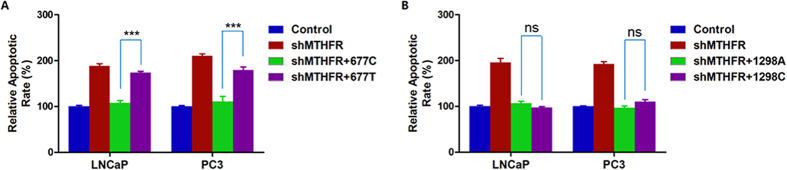
*MTHFR* c.677T variant increases cell apoptosis. (**A**) c.677T variant of *MTHFR* increases cell apoptosis remarkably in both LNCaP and PC3 cell lines. (**B**) c.1298C variant of *MTHFR* has no effect on cell apoptosis. ***Indicates *P* < 0.001, ns indicates no significance.

We tested cell apoptosis in four cell groups (control, *MTHFR*-knockdown, sh*MTHFR* + wild-type *MTHFR* and sh*MTHFR* + c.677T/c.1298C *MTHFR*) in LNCaP and PC3 cell lines, respectively. We found that the c.677T variant of *MTHFR* significantly increased cell apoptosis compared with wild-type genotype in both cell lines (Fig. 4A). However, we did not observe a significant difference between the c.1298C variant and wild-type *MTHFR* overexpressed cell lines (Fig. 4B).

### *MTHFR* c.677T variant inhibits cell proliferation

**Figure 5:**
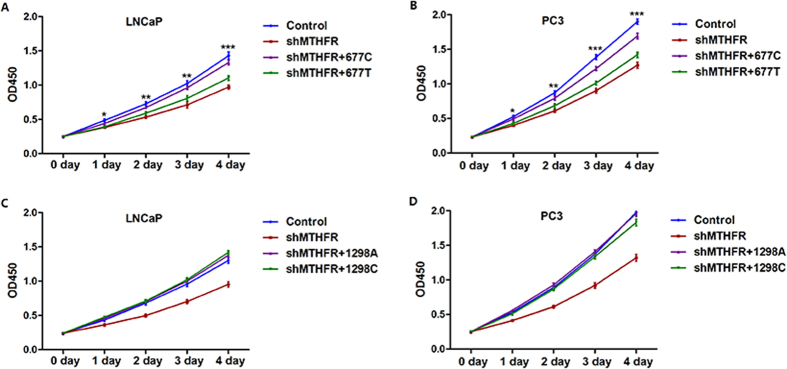
*MTHFR* c.677T variant inhibits cell proliferation. (**A**) c.677T variant of *MTHFR* inhibits cell proliferation significantly compared with wild-type *MTHFR* in LNCaP cells. (**B**) c.677T variant of *MTHFR* inhibits cell proliferation significantly compared with wild-type *MTHFR* in PC3 cells. (**C**) c.1298C variant of *MTHFR* has no significant effect on cell proliferation compared with wild-type *MTHFR* in LNCaP cells. (**D**) c.1298C variant of *MTHFR* has no significant effect on cell proliferation compared with wild-type *MTHFR* in PC3 cells. *Indicates *P* < 0.05, **indicates *P* < 0.01, ***indicates *P* < 0.001, all comparisons were performed between groups of sh*MTHFR* + wild-type *MTHFR* and sh*MTHFR* + c.677T/c.1298C *MTHFR*.

CCK-8 assays were performed to evaluate cell proliferation in the above-mentioned four cell groups in LNCaP and PC3 cell lines, respectively. We found that the c.677T variant of *MTHFR* could inhibit cell proliferation in both cell lines, compared with wild-type *MTHFR* (Fig. 5A,B). In addition, we did not find any effect on cell proliferation in c.1298C variant overexpressed cell lines, compared with wild-type *MTHFR* overexpressed cell lines (Fig. 5C,D).

## Discussion

We performed a large-scale, hospital-based, case-controlled study in Han Chinese men native to Shanghai to determine whether *MTHFR* c.677C>T and c.1298A>C were associated with PCa risk. We found that both heterozygous CT genotype and homozygous TT genotype carriers of rs1801133 had a significantly lower risk of developing PCa compared with those carrying wild-type CC genotype, which is in agreement with previous studies performed in Taiwanese patients^25^ and the Liaoning Province^28^. In addition, after stratification analysis using a recessive model, we found that homozygous TT genotype of rs1801133 was clearly associated with a decreased risk of PCa in patients with Gleason score ≥8, patients with extracapsular extension, patients with seminal vesicle invasion and patients with or without lymph node involvement. Further functional analyses revealed that *MTHFR* c.677T contributed to elevating homocysteine level, increasing DNA damage and cell apoptosis, and inhibiting proliferation of PCa cells.

Variants of *MTHFR*, which is a critical enzyme for intracellular folate homeostasis and metabolism, were reported to be associated with elevated circulating homocysteine levels and many diseases including PCa. Most of the studies focusing on the relationship between PCa and SNPs of *MTHFR* paid close attention to *MTHFR* polymorphism and PCa risk and have conflicting results. A recent meta-analysis which enrolled 22 studies revealed that the *MTHFR* C677T polymorphism did not contribute to the risk of PCa in the overall population of Caucasians, but indicated that it may play a role in PCa development in Asian males^23^. Another recently published meta-analysis which included over 20,000 participants declared that the C677T CT polymorphism of *MTHFR* may be a risk factor of PCa in East Asians, and the association between A1298C variant and PCa risk may vary in different ethnic populations^21^. Clearly, these results indicated that the function of these two common *MTHFR* polymorphisms may vary in different ethnic populations, which suggested that further, large-scale research in different regions and races was needed. Furthermore, among all of the studies enrolled by these meta-analysis publications, only the studies performed by Wu^25^ and Cai^28^ focused on Asian males of Mongoloid race. Wu *et al*. performed studies in Taiwan while Cai *et al*. performed studies in the Liaoning province, regions in different latitudes. It’s reported that activity and variant distribution of *MTHFR* might vary among regions in different latitudes and UV-exposure levels^29^,^30^. On the other hand, ethnic Han Chinese people account for over 92% of the population in China, with more than 1 billion people. Hence, genetic background varied immensely among people in different regions. Furthermore, sample sizes were relatively small in Wu’s and Cai’s research. In the present large-scale study from Shanghai, we confirmed that T allele of rs1801133 may be a protective factor of PCa and rs1801131 may have no significant association with PCa risk.

It has long been recognized that deficiency of folate may increase the incidence of cancer and many other diseases. Deficiency of folate contributes to a higher homocysteine level, thus leading to increased cytosolic calcium and reactive oxygen species (ROS)^31^. Also, DNA damage and chromosome aberrations were induced via ROS-mediated signaling pathways^32^. Moreover, the methylation of cytosine in DNA will be reduced when there is a folate deficiency, and will eventually cause an aberrant expression of pro-oncogene and a potential malignant transformation in carcinogenesis^33^. *MTHFR* is directly involved in the metabolism of folate and is considered to have direct consequences to cancer incidence if the enzymatic function is partly lost. It’s reported that the homozygous status of these two common variants of the *MTHFR* gene (rs1801133 and rs1801131) will cause about a 70% and 40% reduction of enzyme function compared with wild-type genotype, respectively^20^. Therefore, it was ironic to find that T allele of C677T polymorphism of the *MTHFR* gene was a protective factor of PCa risk. However, some research groups found that the 677TT genotype of the *MTHFR* gene might allow the expression of tumor suppressor genes and reduce the risk of cancer development while it partly caused enzymatic dysfunction^34^. Furthermore, *MTHFR* inhibition was proven to arrest the growth of cancer cells in *in vitro* studies because of limited methionine supply^35^. Cell apoptosis could also be induced by an increased ROS level and could play a favorable role in cancer cells^36^. These findings may partly support our results, and we still need more basic, functional research to determine the exact function of *MTHFR* variants in PCa carcinogenesis, development, and progression.

We acknowledged that there were some limitations in our study design and performance. Firstly, there may be some biases from samples collection in this hospital-based, case-controlled study. Although these biases could be minimized by taking into consideration confounding factors and age-matching between cases and controls. Secondly, in the stratified analysis, the case number in subgroups was relatively small and it would decrease the statistical power to examine the genetic variants with regard to PCa risk. Thirdly, folate intake, which is an important factor affecting the function of *MTHFR* polymorphism on PCa risk, was another condition not available from participants. Finally, potential mechanisms of the long-distance regulatory effect of the two studied *MTHFR* polymorphisms on the expression of *MTHFR* was not explored in-depth, which warrants further investigation in our future study. Notwithstanding these limitations, our research underscores that ethnic Han Chinese males carrying *MTHFR* c.667T allele had a significantly lower risk for developing PCa compared with those with wild-type *MTHFR* genotype of rs1801133. Further functional analyses revealed that the *MTHFR* c.677T contributes to elevating homocysteine level, increasing DNA damage and cell apoptosis, and inhibiting proliferation of PCa cells, thereby decreasing PCa risk.

In conclusion, our findings indicated that *MTHFR* c.677T variant acts as a protective factor of PCa risk by elevating homocysteine level, increasing DNA damage and cell apoptosis, and inhibiting proliferation of PCa cells. Further research focusing on gene-gene or gene-environment interactions should be conducted to explore more legible mechanisms of the role *MTHFR* plays in PCa risk.

## Materials and Methods

### Study population

This hospital-based, case-controlled study recruited 1817 newly diagnosed PCa patients, and 2026 matched cancer-null controls from genetically unrelated ethnic Han Chinese men treated at Fudan University Shanghai Cancer Center from January 2008 to June 2015. Two well-trained pathologists, specializing in genitourinary cancer, assessed the histological type of the PCa patients independently. All of the cases were defined as primary prostate adenocarcinoma according to the WHO criteria for PCa. We excluded patients with malignancies other than primary PCa, patients with a family history of PCa, and patients who received chemotherapy or radiotherapy before enrollment. Tumor stage was assessed and classified according to the TNM classification system provided by the American Joint Committee on Cancer (AJCC) in 2010^37^. The Gleason score system was employed to determine histological grading of the PCa patients. We collected patients’ clinical and pathological information at the time they were enrolled, including general clinical features (age at diagnosis, height, weight, circulating PSA level), characteristics at surgery (tumor grade, tumor stage, surgical margin status and lymph node involvement) and comorbidities (hypertension, cardiovascular disease and diabetes mellitus).

Frequency-match was performed in the 2026 cancer-null Chinese men by age at diagnosis and geographic area according to the PCa patients’ characteristics. Since serum PSA screening and digital rectal examination were not routinely performed in China, we advised people with urinary tract symptoms to have a serum PSA measurement and a digital rectal examination. Participants with a serum PSA level >4 ng/ml, with or without an abnormal digital rectal examination, were also excluded from the control database.

All of the study designs and testing procedures were performed according to the ethical standards of the Helsinki Declaration II and approved by the Scientific and Ethical Committee of Fudan University Shanghai Cancer Center. Written informed consents were obtained from all participants before any study-specific investigation was performed.

### SNP identification and genotyping

We extracted genomic DNA from peripheral leukocytes using the Qiagen Blood DNA Mini Kit (Qiagen Inc., Valencia, CA, USA) following the standard protocols. Our selected SNPs of *MTHFR* gene, rs1801133 (NM_005957.4:c.665C>T; also known as C677T) and rs1801131 (NM_005957.4:c.1286A>C; also known as A1298T), were all located in exon region and were genotyped by SNaPshot analysis (ABI). Among all of the testing results, we used direct dye terminator sequencing of PCR to validate 5% of them according to the manufacturer’s instructions of ABI Prism BigDye system (ABI, Foster City, CA, USA). The samples for sequencing and genotyping were run on an ABI 3730 automated sequencer and analyzed by SeqMan and Peakscan, respectively.

### Cell lines and cell culture

Human LNCaP and PC3 cell lines (The Cell Bank of Chinese Academy of Sciences, Shanghai) were cultured in RPMI 1640 media (Invitrogen, Carlsbad, CA) supplemented with 10% fetal bovine serum (Invitrogen). The cells were maintained in 5% CO_2_ at 37 °C and the media was replaced every other day.

### Plasmid construction, mutation genesis, and transfection

Full-length *MTHFR* cDNA was amplified by PCR, and CDS was cloned into the pcDNA3.1 vector (Invitrogen, Carlsbad, CA, USA). The corresponding C677T and A1298C mutated plasmid was generated by site-directed mutagenesis with the MutanBEST Kit (Takara, Berkeley, CA) to ensure a uniform backbone sequence. We verified all recombinant clones by DNA sequence. The expression plasmids were transfected into cells using Lipofectamine 2000 (Invitrogen) according to the manufacturer’s instructions.

### Western blot

Standard procedures were followed for western blot analysis. Antibodies used for western blotting included anti-*MTHFR* (Cat NO. ab203786, abcam) and anti-β-ACTIN (Cat NO. A00702, Gensctipt).

### RNAi silencing of *MTHFR*

Lentiviral vector (pLKO.1) expressing shRNA clones were generated by the Stealth RNAi^TM^ siRNAs platform. The shRNA against 3′ UTR of human *MTHFR* gene was generated using the following target sequence: 5′-CAGTGGCAGTGAGAGCTCCAAAGAT-3′.

1 × 10^7^ cells were seeded in a 10 cm dish with 7 ml viral medium containing 8 μg/ml Polybrene for lentiviral infection. Cells were selected for infection by the addition of 2 μg/ml puromycin after 24 hours. Uninfected cells demonstrated 100% cell death after cultured with puromycin within 3 days.

### Measurement of serum and cellular homocysteine level

We randomly selected 306 controls to test the circulating homocysteine level. EDTA-plasma samples were collected, centrifuged immediately, and stored at −80 °C for analyzing. Plasma and cellular homocysteine levels were detected using a tHcy Detection kit (Kuake Biotechnology, Zhejiang, China) by AU5800 biochemistry analyzer (Beckman Coulter, California, USA) according to the manufacturer’s protocol.

### Comet Assay for DNA damage

The Comet Assay Kit (Trevigen, Gaithersburg, MD) was used to detect single- and double-stranded DNA breaks in cultured cells. Slides were viewed (excitation 425–500 nm) with a Leica DMI 4000B epifluorescence microscope. These slides were used for each condition. In normal cells, the fluorescence is confined mostly to the nucleus because undamaged DNA cannot migrate. In cells with DNA damage, DNA is denatured by the alkali solution used for single-strand break detection, or the neutral solution used for double-strand break detection; and the negatively charged DNA fragments are released from the nucleus and migrate toward the anode.

### Cell apoptosis detection

Cells were harvested and stained with Annexin V-FITC and propidium iodide (Annexin V-FITC apoptosis detection kit, B.D. Biosciences Pharmingen, San Jose, CA, USA) for 15 minutes at room temperature and protected from light. Finally, the cell suspension was filtered by a nylon sieve and analyzed by an Accuri C6 flow cytometer (BD Biosciences).

### Cell proliferation assay

Cell proliferation after transfection was measured using the Cell Counting Kit-8 (Dojindo Laboratories, Kumamoto, Japan). In brief, LNCaP and PC3 cells were seeded in 96-well plates and allowed to adhere. A 10 μl CCK-8 solution was added to each well and incubated in a humidified CO_2_ incubator at 37 °C for 2 hours. Then, samples taken from each well were measured at 450 nm, and the percentage of surviving cells in each treatment group was plotted relative to the untreated one.

### Statistical analysis

The body mass index (BMI, kg/m^2^) was calculated as the weight in kilograms divided by the height in square meters. We used the WHO cut point as an index for defining overweight status^38^ (BMI ≥ 25 kg/m^2^) in the Asian population. Continuous variables were presented as mean ± standard deviation (SD) and categorical variables were reported in the form of a number (proportion).

The goodness-of fit χ^2^ test was performed to calculate Hardy-Weinberg equilibrium (HWE) for evaluating genotype distribution in control subjects, and *P* value < 0.05 was considered deviated from equilibrium. We used univariate and multivariate unconditional logistic regression models to calculate crude and adjusted odds ratios (ORs) and 95% confidence intervals (CIs), respectively, to assess associations between the genotypes and PCa risk. In a multivariate model, covariants included age at diagnosis, BMI, hypertension, diabetes mellitus, and cardiovascular disease. Furthermore, we performed a stratified analysis to explore the relationship between the genotypes and risk of PCa among subgroups of age at diagnosis (≤68 vs. >68), BMI (<25 vs. ≥25), hypertension, diabetes mellitus, cardiovascular disease, Gleason score, extracapsular extension, seminal vesicle invasion, positive surgical margin and lymph node involvement. We used Chi-square-based Q test to evaluate the homogeneity of associations between subgroups. In all statistical analyses, a two-sided *P* < 0.05 was viewed as significant. We performed all statistical analyses using SPSS software version 16.0 (SPSS Inc., Chicago, IL, USA).

## Additional Information

**How to cite this article**: Wu, J.-L. *et al*. *MTHFR* c.677C>T Inhibits Cell Proliferation and Decreases Prostate Cancer Susceptibility in the Han Chinese Population in Shanghai. *Sci. Rep*. **6**, 36290; doi: 10.1038/srep36290 (2016).

**Publisher’s note:** Springer Nature remains neutral with regard to jurisdictional claims in published maps and institutional affiliations.

## Supplementary Material

Supplementary Information

## Figures and Tables

**Table 1:** Distribution of demographic and clinicopathological characteristics of 1817 PCa patients and 2026 controls included in the study.

Variables	Cases (n = 1817)	Controls (n = 2026)	*P*
Age (yr), mean ± SD	66.7 ± 7.2	66.9 ± 6.8	0.437
BMI (kg/m2), n (%)			0.877
<25	1308 (72.0)	1463 (72.2)	
≥25	509 (28.0)	563 (27.8)	
Hypertension, n (%)			0.424
No	1054 (58.0)	1201 (59.3)	
Yes	763 (42.0)	825 (40.7)	
Cardiovascular disease, n (%)			0.631
No	1660 (91.4)	1842 (90.9)	
Yes	157 (8.6)	184 (9.1)	
Diabetes mellitus, n (%)			0.926
No	1636 (90.0)	1826 (90.1)	
Yes	181 (10.0)	200 (9.9)	
PSA (ng/mL), mean ± SD	28.5 ± 1.3	1.2 ± 0.3	<0.001
Gleason score, n (%)			
≤6	289 (15.9)		
7	923 (50.8)		
≥8	605 (33.3)		
Pathological tumor stage, n (%)			
T2	1231 (67.7)		
T3a	160 (8.8)		
T3b	426 (23.4)		
Lymph node involvement, n (%)	154 (8.5)		
Positive surgical margins, n (%)	352 (19.4)		

PCa, prostate cancer.

**Table 2:** Association between genetic polymorphisms in folate metabolism genes and PCa risk in Han Chinese men.

Gene	SNP	Type	Genotype	Cases (n = 1817)	Controls (n = 2026)	*P*^HWE^	Crude OR (95% CI)	*P*	Adjusted OR (95% CI)^c^	*P*^a^
MTHFR	rs1801133	Nonsynonymous (exon 4)	CC	654 (36.0)	599 (29.6)	0.580	1.00	**1 × 10**^**−5**^	1.00	**3 × 10**^**−4**^
			CT	876 (48.2)	1022 (50.4)		**0.79 (0.68–0.91)**		**0.78 (0.67–0.92)**	
			TT	287 (15.8)	405 (20.0)		**0.65 (0.54–0.78)**		**0.68 (0.55–0.83)**	
			Dominant model				**0.75 (0.65–0.85)**	**2 × 10**^**−5**^	**0.75 (0.65–0.87)**	**2 × 10**^**−4**^
			Recessive model				**0.75 (0.64–0.89)**	**7 × 10**^**−4**^	**0.78 (0.65–0.94)**	**0.008**
			Additive model				**0.80 (0.73–0.88)**	**6 × 10**^**−5**^	**0.82 (0.74–0.90)**	**1 × 10**^**−4**^
	rs1801131	Nonsynonymous (exon 7)	AA	1192 (65.6)	1355 (66.9)	0.220	1.00	0.690	1.00	0.680
			AC	569 (31.3)	609 (30.1)		1.06 (0.92–1.22)		1.07 (0.92–1.24)	
			CC	56 (3.1)	62 (3.1)		1.03 (0.71–1.49)		1.06 (0.72–1.58)	
			Dominant model				1.06 (0.93–1.21)	0.400	1.07 (0.92–1.24)	0.380
			Recessive model				1.01 (0.70–1.45)	0.970	1.04 (0.70–1.55)	0.830
			Additive model				1.05 (0.93–1.17)	0.460	1.06 (0.93–1.20)	0.410

PCa, prostate cancer; OR, odds ratio; 95%CI, 95% confidence interval.

^HWE^*P* value for the Hardy–Weinberg equilibrium test in controls subjects.

^a^Adjusted for age, BMI, hypertension, diabetes mellitus and cardiovascular disease in multivariant logistic regression models.

**Table 3:** Stratified analysis for associations between genetic polymorphisms in folate metabolism genes and PCa risk by recessive genetic model in Han Chinese men.

Variables	rs1801133 (cases/controls)		Adjusted OR^a^ (95% CI)	*P*	*P*^hom^	rs1801131 (cases/controls)		Adjusted OR^a^ (95% CI)	*P*	*P*^hom^
	CC/CT	TT				AA/AC	CC			
Age (yr), median										
≤68	850/1053	167/252	0.86 (0.67–1.11)	0.250	0.194	985/1260	32/45	0.93 (0.53–1.61)	0.780	0.390
>68	680/568	120/153	**0.67 (0.48–0.92)**	**0.014**		776/704	24/17	1.19 (0.57–2.52)	0.640	
BMI (kg/m2)										
<25	1112/1178	196/285	**0.73 (0.60–0.89)**	**0.002**	0.596	1263/1424	45/39	1.30 (0.84–2.02)	0.230	**0.034**
≥25	418/443	91/120	0.82 (0.60–1.11)	0.190		498/540	11/23	0.53 (0.25–1.09)	0.076	
Hypertension										
No	887/975	167/226	0.87 (0.68–1.11)	0.250	0.276	1023/1166	31/35	0.94 (0.54–1.64)	0.830	0.982
Yes	643/646	120/179	**0.66 (0.49–0.90)**	**0.007**		738/798	25/27	1.34 (0.73–2.45)	0.340	
Diabetes mellitus										
No	1382/1467	254/359	**0.77 (0.63–0.94)**	**0.008**	0.982	1584/1771	52/55	1.07 (0.71–1.63)	0.740	0.427
Yes	148/154	33/46	0.78 (0.43–1.42)	0.410		177/193	4/7	0.55 (0.13–2.40)	0.420	
Cardiovascular disease										
No	1399/1483	261/359	**0.80 (0.66–0.98)**	**0.027**	0.671	1611/1783	49/59	0.92 (0.61–1.40)	0.700	0.123
Yes	131/150	26/34	0.73 (0.35–1.52)	0.390		150/181	7/3	**6.49 (1.08–39.01)**	**0.028**	
Gleason score										
≤7	997/1621	215/405	0.94 (0.76–1.16)	0.560	**0.005**	1177/1964	35/62	1.05 (0.66–1.68)	0.820	0.570
≥8	533/1621	72/405	**0.53 (0.39–0.71)**	**3 × 10**^**−5**^		584/1964	21/62	1.03 (0.57–1.84)	0.930	
Extracapsular extension										
No	1018/1621	213/405	0.94 (0.77–1.16)	0.590	**0.025**	1194/1964	37/62	1.09 (0.68–1.74)	0.720	0.818
Yes	512/1621	74/405	**0.51 (0.38–0.70)**	**8 × 10**^**−5**^		567/1964	19/62	1.10 (0.61–2.00)	0.750	
Seminal vesicle invasion										
No	1154/1621	237/405	0.92 (0.76–1.12)	0.420	**0.018**	1351/1964	40/62	1.03 (0.65–1.61)	0.910	0.433
Yes	376/1621	50/405	**0.43 (0.29–0.62)**	**2 × 10**^**−5**^		410/1964	16/62	1.21 (0.63–2.32)	0.560	
Positive surgical margin										
No	1234/1621	231/405	**0.82 (0.67–1.00)**	**0.045**	0.953	1419/1964	46/62	1.02 (0.67–1.56)	0.920	0.796
Yes	296/1621	56/405	**0.69 (0.47–1.00)**	**0.046**		342/1964	10/62	1.27 (0.57–2.82)	0.570	
Lymph node involvement										
No	1382/1621	281/405	**0.87 (0.72–1.04)**	**0.130**	**<0.001**	1616/1964	47/62	0.92 (0.61–1.40)	0.700	0.067
Yes	148/1621	6/405	**0.13 (0.05–0.32)**	**1 × 10**^**−6**^		145/1964	9/62	**3.38 (1.41–8.13)**	**0.011**	

PCa, prostate cancer; OR, odds ratio; 95%CI, 95% confidence interval.

^a^Adjusted for age, BMI, hypertension, diabetes mellitus, and cardiovascular disease in multivariant logistic regression models.

^hom^*P* value for homogeneity test using the χ^2^-based Q-test.
