# Supplementary material for: MTHFR c.677C>T Inhibits Cell Proliferation and Decreases Prostate Cancer Susceptibility in the Han Chinese Population in Shanghai
Source: Sci Rep. 2016 Nov 7;6:36290. doi: 10.1038/srep36290 (PMC5098242; doi:10.1038/srep36290)
Supplement: Supplementary Information [file srep36290-s1.pdf]

Supplementary file

MTHFR c.677C>T Inhibits Cell Proliferation and Decreases Prostate Cancer Susceptibility in the Han Chinese Population in Shanghai

Running head: *MTHFR* c.677C>T decreases PCa susceptibility

Jun-Long Wu[1,5,†], Shu-Xian Zhou[2,3,†], Rui Zhao[2,3], Xuan Zhang[2], Kun Chang[1,5], Cheng-Yuan Gu[1,5], Hua-Lei Gan[4,5], Bo Dai[1,5], Yao Zhu[1,5], Hai-Liang Zhang[1,5], Guo-Hai Shi[1,5], Yuan-Yuan Qu[1,5*], Jian-Yuan Zhao[2,*], Ding-Wei Ye[1,5*]

[†]These authors contributed equally to this work.

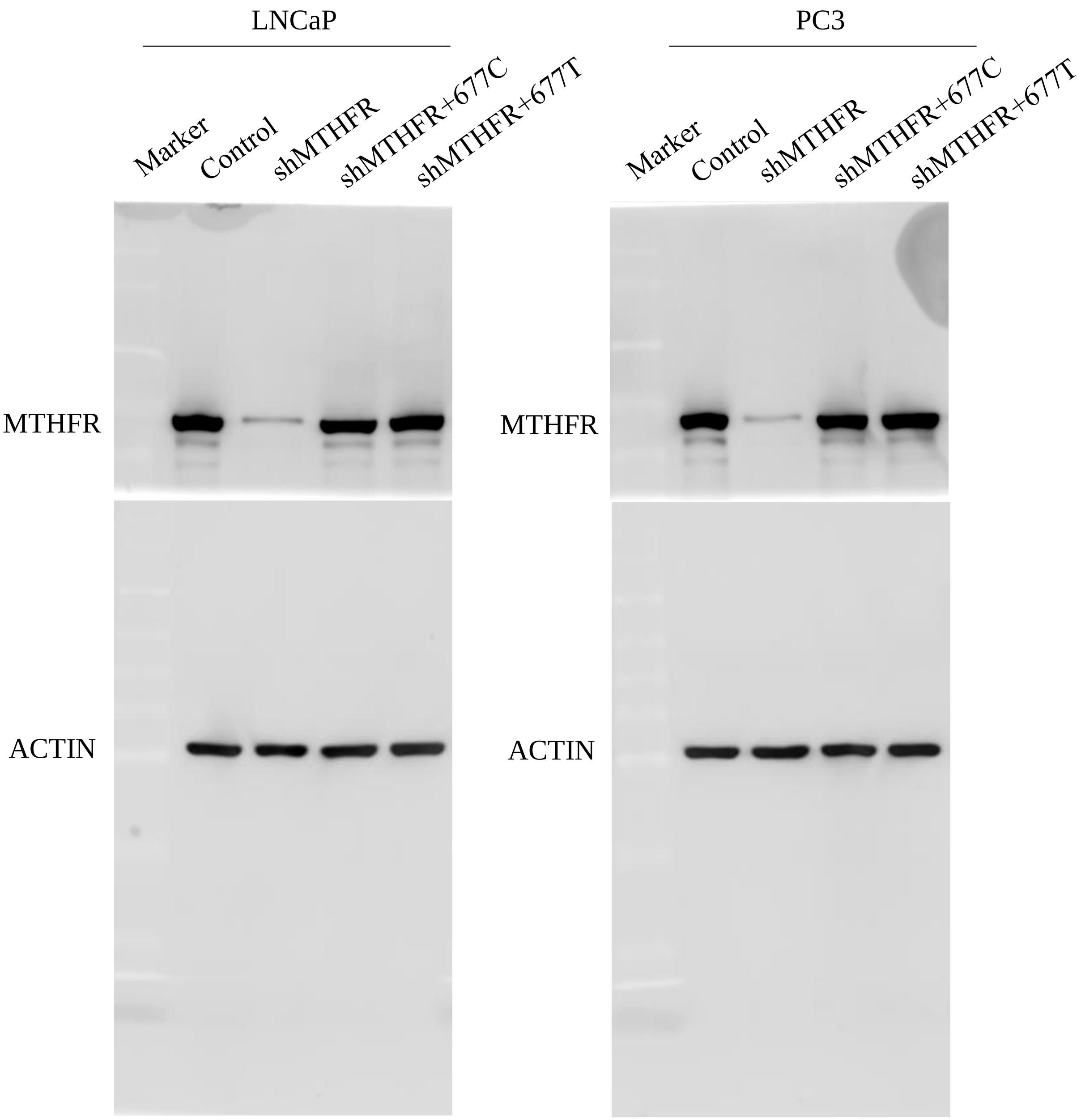

Supplementary Figure 1. The full-length blots for knockdown efficiency of *shMTHFR* and protein expression of MTHFR after restoration of wild-type or mutant *MTHFR* gene measured by western blot.
